# Addressing the impact of economic sanctions on Iranian drug shortages in the joint comprehensive plan of action: promoting access to medicines and health diplomacy

**DOI:** 10.1186/s12992-016-0168-6

**Published:** 2016-06-08

**Authors:** Sogol Setayesh, Tim K. Mackey

**Affiliations:** Joint Masters Degree Program in Health Policy and Law, University of California, San Diego School of Medicine – California Western School of Law, San Diego, CA USA; Department of Anesthesiology, University of California, San Diego School of Medicine, San Diego, CA USA; Department of Medicine, Division of Global Public Health, University of California, San Diego School of Medicine, San Diego, CA USA; Global Health Policy Institute, 6256 Greenwich Dr., Room 137, San Diego, CA 92122 USA

**Keywords:** Iran, Economic sanctions, Medical shortage, Humanitarian crisis, Health diplomacy, Human rights

## Abstract

**Background:**

The U.S Congress initiated sanctions against Iran after the 1979 U.S. Embassy hostage crisis in Tehran, and since then the scope of multilateral sanctions imposed by the United States, the European Union, and the United Nations Security Council have progressively expanded throughout the intervening years. Though primarily targeted at Iran’s nuclear proliferation activities, sanctions have nevertheless resulted in negative public health outcomes for ordinary Iranian citizens. This includes creating vital domestic shortages to life-saving medicines, leaving an estimated 6 million Iranian patients with limited treatment access for a host of diseases. Sanctions have also crippled Iran’s domestic pharmaceutical industry, leading to the disruption of generic medicines production and forcing the country to import medicines and raw materials that are of lower or questionable quality.

**Discussion:**

Countries such as the United States have responded to this medical crisis by implementing export control exemptions with the aim of easing the trade of humanitarian goods (including certain pharmaceuticals and medical devices). However, despite these efforts, pharmaceutical firms and international banking institutions remain cautious about doing business with Iran, leaving the country faced with continuing shortages. We conducted a review of key characteristics of the Iranian drug shortage that identified 73 shortage drugs that closely tracked with the disease burden in the country. Additionally, 44 % of these drugs were also classified as essential medicines by the World Health Organization. A vast majority of these drugs were also covered under export control exemptions that theoretically should make them easier to procure, but nevertheless will still in shortage.

**Summary:**

Based on our review of the sanctions regulatory framework and key characteristics of the Iranian drug shortage, we propose policy intervention leveraging the recently negotiated P5 + 1 agreement that begins the process of providing Iran relief from the international economic sanctions regime. This specifically includes advocating for the application of “health diplomacy” in ongoing multilateral negotiations following commencement of “implementation day,” by advocating for an additional set of reform measures incorporated into this historic negotiation that will finally address the humanitarian and medical crisis of drug shortages in Iran.

## Background

On October 18, 2015, the United States approved a set of conditional sanction waivers for the Islamic Republic of Iran following agreement on the historic Joint Comprehensive Plan of Action (JCPOA) reached by the P5 + 1 (comprised of the five permanent members of the UN Security Council and Germany) in July 2015 [1]. On January 16, 2016, this agreement reached a critical milestone, with the International Atomic Agency (IAEA) certifying that Iran had successfully complied with a set of JCPOA nuclear dismantlement requirements, triggering commencement of “Implementation Day.”

Importantly, “Implementation Day” marks a critical first step towards the lifting of a host of multilateral nuclear-related sanctions imposed by the United States, the European Union (EU), the United Nations Security Council, and other countries, that had been increasing in scope and have crippled the Iranian economy for more than three decades. Compliance to the terms of the JCOPA represents a possible end to Iran’s nuclear weapons ambitions and also marks the beginning of a period of transition that will reintegrate Iran back into the global economy [1, 2]. However, overlooked in this landmark development in foreign policy and diplomacy is an unresolved public health crisis directly related to economic sanctions: an ongoing critical domestic medicines shortage in Iran.

Although the primary intentions of the historically imposed Iranian sanction regime were to limit economic development, international trade, and scientific and military assistance, sanctions have also directly contributed to creating critical medicine shortages within the country that persist to this day [3]. This is despite the fact that revisions have been made to export controls by the United States (U.S.) Office of Foreign Assets Control (OFAC) in an attempt to better facilitate humanitarian trade [4]. Despite these efforts, Iran continues to face ongoing challenges to importing life-saving medicines, supplies, and medical raw materials from the United States and EU [4, 5]. This has resulted in an estimated 6 million Iranian patients who lack access to essential treatment needed to address highly prevalent communicable and non-communicable diseases (NCDs) within the country [6]. The once self-sufficient domestic pharmaceutical industry has also had its production of generic drugs severely disrupted, a situation that has introduced risks to patients, including the detection of substandard and counterfeit medicines [7, 8].

In response, this debate piece conducts a review of the history, regulatory export policies, public health impacts, and identifies key characteristics associated with the Iranian medicines shortage. We first examine the history of Iranian sanctions and how it has influenced trade and financial regulatory responses that have directly contributed to the medicines shortage. We then assess the public health impact of the economic sanctions regime by identifying and characterizing the types of drugs currently in shortage. Finally, the article concludes with a policy proposal designed to take advantage of the historic JCPOA agreement specifically advocating for the application of the concept of “health diplomacy.” This would be accomplished by introducing a set of policy interventions that could be included in ongoing implementation of the JCPOA aimed at ensuring the protection of health and human rights of the Iranian people by promoting equitable and safe access to medicines.

## Breif history of the Iranian economic sanctions regime

**Fig. 1:**
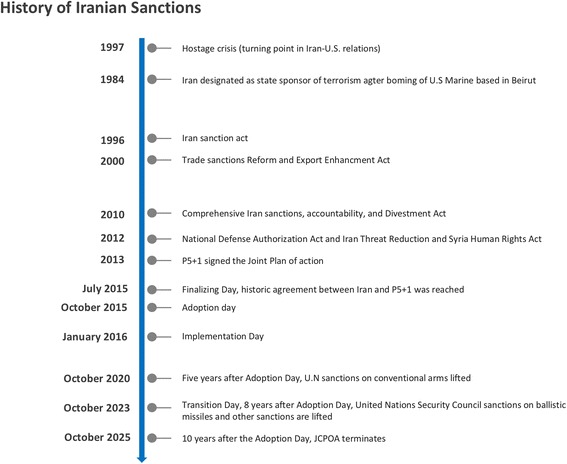
History of Iran sanctions

The decades-long comprehensive unilateral and multilateral Iranian economic sanctions regime led by the United States, the European Union, the UN Security Council, and several other countries, has historically focused on resolving the nuclear impasse between Iran and the West. The origin of U.S.-led sanctions against Iran dates as far back as 1967, when Iran acceded to the Non-Proliferation Treaty (NPT) and later in 1974 to the IAEA Safeguards Agreement, both of which required non-nuclear weapon states to agree to international norms of nonproliferation, peaceful uses of nuclear energy, disarmament, and consent to IAEA inspections [3]. During intervening years, domestic political upheaval followed, and Iran was accused of restarting its nuclear program in violation of its treaty obligations, which led to a series of economic sanctions [3]. This included sanctions imposed by the U.S. government, which passed legislation and issued executive orders that have crippled Iran’s economy, oil exports, and also weakened its public health system [9, 10] (see Fig. 1 for timeline).

The first of a series of U.S. sanctions were imposed in 1979, when then U.S. President Jimmy Carter ordered a freezing of Iranian assets in response to U.S. diplomats being held hostage at the U.S. Embassy in Tehran, a turning point in U.S.–Iranian relations [3]. Additional sanctions were imposed in 1984 when Iran was designated a state sponsor of terrorism, in the wake of Hezbollah’s bombing of a marine base in Beirut, leading to an embargo of Iranian crude oil imports followed by sanctions on all Iranian goods [3]. Western powers imposed further unilateral sanctions from 1996–2006, starting with the United States’ enactment of the Iran Sanctions Act (ISA) that prohibited investment of over $20 million per annum by foreign companies in Iran’s energy sector. From 2006–2010, sanctions were also pursued multilaterally, with the UN Security Council adopting several resolutions in response to international concerns about Iran’s nuclear weapons ambitions and its continued operation of its nuclear program [3, 10].

In 2010, the United States enacted a set of new sanctions as part of the Comprehensive Iran Sanctions, Accountability, and Divestment Act (CISADA) that targeted energy-sector activities, including insurance and shipping companies involved with Iranian imports and exports [11]. From 2011–2012, the U.S. Congress also voted in favor of a series of new and unprecedented sanctions against Iran’s financial sector, specifically its Central Bank, which barred companies and countries from doing business with Iranian financial institutions, implemented as part of the specifications in the National Defense Authorization Act and the Iran Threat Reduction and Syria Human Rights Act [11, 12].

These financial sanctions were aimed at blocking Iran’s access to the global banking system and the Society for Worldwide Interbank Financial Telecommunication (SWIFT), which serves 212 countries and provides secure message exchange services for over 10,000 banking organizations [13]. Discontinuing Iran’s SWIFT access meant all transactions became both expensive and lengthy [4]. These sanctions also placed certain Iranian banks on the OFAC’s Specially Designated Nationals List (SDN List), which identifies individuals and entities prohibited from accessing the U.S. financial system [14]. Eventually, these banking sanctions would expand to over 16 Iranian banks and the Islamic Revolutionary Guard, all entities heavily involved in the Iranian economy [15].

In addition to economic sanctions imposed by the United States, the European Union has utilized a series of regulations and sanctions that have arguably had a greater negative impact on Iran’s economy given the large volume of shared trade and economic activity shared between the regions [16]. Council of the European Union sanctions that came into effect in July 2010 targeted specific Iranian companies, froze assets, and suspended economic activity, with a primary focus on sectors of energy, insurance, transport, finance, and aviation. These sanctions went beyond implementation of UN-mandated sanctions, instead imposing what has been termed as a set of “comprehensive restrictive measures” that have played a key role in damaging Iran’s economy and disrupting key trade activities, including medicines procurement [16].

## The economic impact of sanctions on Iran

Crucially, the impact of collective sanctions imposed by the US, the EU, and other countries, has made exporting and importing to and from Iran extremely challenging, primarily due to potential trading partners’ inability to transfer money to Iran in foreign currency and vice versa. This forces the Iranian government and domestic companies to either accept the trading partner’s national currency or to barter for other goods [4, 17, 18]. Accordingly, many companies have ceased doing business with the Iran all together, fearing reprisal for sanction violations.

For example, several leading international financial institutions, including Swiss financial firm Credit Suisse and London-based Standard Chartered Bank, incurred hundreds of millions of dollars in fines for sanctions violations for trading with Iran [5]. In December 2012, the U.K.’s Standard Chartered Bank paid a $327 million penalty to the U.S. Department of the Treasury because it had conducted 60,000 transactions in the interest of Iranian financial institutions [19]. Other institutions also have been the subject of Iranian sanction violation investigations, including the Royal Bank of Scotland, UniCredit, HSBC, Deutsche Boerse, Société Générale, and Crédit Agricole [20].

International sanctions have also contributed to a compromised and declining Iranian economy, though other factors such as economic mismanagement, corruption, and trepidation by investors due to political instability and potential military conflict have also taken their toll [16]. For example, Iran’s GDP shrank by 9 % and its overall economy is 15 to 20 % smaller since the latest round of sanctions in 2012 [2]. Since 2010, the Iranian currency has destabilized and lost two thirds of its value against the U.S. dollar, marked by an inflation rate of 40–50 % in 2013 [15, 21]. Iran has also lost $160 billion in oil revenue, and more than $100 billion in Iranian assets were frozen in foreign financial institutions [2]. This significant drop in energy-related revenue is pivotal because the national economy is so highly dependent on the oil industry as part of its export economy, which under the sanctions regime has been heavily embargoed [15, 22].

Successive waves of sanctions have also led to a dramatic increase in the cost of doing business in Iran. Circumventing sanctions to carry out customary business and trading activities costs Iranian companies millions of dollars because businesses must find alternative ways to transfer money and ship goods [23]. To address the inherent risk of doing business with Iran, some foreign banks charge fees as high as 5 % to transfer money in and out of the country and, in some cases, Iranian businesses have to pay middlemen to produce documents that show an origin or destination other than Iran [24].

Most importantly, sanctions have also had a substantial and negative impact on Iran’s public health programs and institutions [25, 26]. According to the World Bank, per capita health expenditures in Iran were last reported (in 2013) at US $432; conversely per-capita health expenditures before the latest round of sanction (in 2012) were reported as US $485. The negative trend illustrates a potential direct relationship between loss of oil revenues and corresponding declines in the country’s ability to invest in national health expenditures. Declines in health expenditures act to exacerbate critical medicine shortages, as economic sanctions make it extremely difficult to obtain medicines, medical supplies and medical devices from abroad and also inhibit the ability to finance the cost of procurement.

## Legal and regulatory framework impacting medicine access

Although ratcheting up of Iranian economic sanctions has intensified due to geopolitical factors and nuclear diplomacy, the need to ease trade of humanitarian goods, including certain medical products, has not gone unnoticed. Prior to October 2012, under the U.S. Trade Sanctions Reform and Export Enhancement Act of 2000 (TSRA), exporters were required to apply for a specific license issued by OFAC (which enforces economic and trade sanctions for the U.S.) to export pharmaceuticals and medical devices to Iran [27]. Stringent requirements for export licensure made it administratively difficult to export medical products, because each product required a specific classification determination by the Bureau of Industry and Security (BIS) under Export Administration Regulations (EAR) [28].

In October 2012, OFAC published new provisions that amended most of the Iranian Transactions Regulations. The renamed Iranian Transactions and Sanctions Regulations (ITSR) clarified the CISADA provisions and further tightened sanctions by blocking the property of Iran’s government and financial institutions [29]. However, in these revisions, OFAC also recognized the humanitarian challenges associated with sanctions and revised the regulations with the aim of easing export of certain pharmaceuticals and medical devices. This resulted in OFAC adding a general license category (i.e., EAR99) that specifically authorized export or re-export of most medicines and medical supplies by allowing items that previously required a specific TSRA license to be exported under a general license that is easier for exporters to obtain [29]. Hence, currently pharmaceutical products are grouped into two different categories: “EAR99” (easier to export as they fall under a “general license”) and “Non-EAR99” (harder to export as additional export controls are required for an export license) drugs (see Table 1).

**Table 1:** EAR99 classified drugs vs. non-EAR99 classified drugs

EAR99	Non-EAR99
(Easier to export)	(Harder to export)
General export license	Specific export license
Not identified on commerce control list	Identified on commerce control list
No export control classification number	Export control classification number
Most medicines, including over-the-counter items, are considered EAR99	Non-NSAID analgesics, cholinergics, anticholinergics, opiods, narcotics, benzodiazipine and and bioactive peptide, vaccines, “immunotoxins” (antibody–toxin conjugates intended to destroy specific target cells such as tumor cells that bear antigens homologous to the antibody), certain toxin-containing medical products and diagnostics, food testing kits, certain medical devices and medical devices parts controlled under export control classification number

Subsequently, in July 2013, OFAC significantly expanded its list of basic medical supplies and issued a set of clarifying guidelines for the sale of medicine and medical devices [30]. These new exportation guidelines exempted transactions for medical export payments by foreign banks that hold Iranian oil revenues, which were previously prohibited under the Iranian financial sanctions regulations. Changes from the ITSR technically had the effect of authorizing the humanitarian sale of a specific subset of medical products to Iran by a U.S. person or companies. The intent of the revised OFAC guidance was to provide regulatory clarity and facilitate trade in humanitarian goods, including for U.S. entities.

However, not all medicines qualify under OFAC’s 2013 revised guidelines. The TSRA defines the terms “medicine” and “medical devices” by the definitions of “drug” and “device” in section 201 of the Federal Food, Drug, and Cosmetic Act (FFDCA) (21 U.S.C. 321) [27]. These definitions include prescription and over-the-counter medicines and medical devices, most of which are classified as EAR99 and hence should be easier to export. However, certain vaccines, biological and chemical products, and medical devices (including medical supplies, instruments, equipment, equipped ambulances, institutional washing machines for sterilization, and vehicles carrying medical testing equipment) are specifically classified as Non-EAR99, and therefore are more difficult or cannot be exported to Iran [27].

The rationale for controlling Non-EAR99–classified drugs is to ensure that transferring certain chemicals, pathogens, toxins, dual-use chemicals, and biological facilities and equipment does not contribute to Chemical and Biological Warfare proliferation. Vaccines, for instance, are integral to public health and are a legitimate and essential pharmaceutical commodity, but may also be used to illegitimately produce a biological weapon, agent, or toxin [28]. Similar concerns have also arisen in the Syrian conflict, where chlorine has been used as a chemical weapon against civilians, while also representing a key commodity in water purification, sanitation, and medicines manufacture [31].

## Impact of sanctions on medicines access in Iran

### Public health impact

Although the revised and current Iranian sanctions regime does not specifically prohibit the export of humanitarian goods and pharmaceuticals, many of the administrative and regulatory processes have made it difficult to export life-saving medicines to Iran. This includes the need to navigate a complex export control regulatory process, the inability of Iranian banks to do business with the international banking system and U.S. corporations, currency shortages, and the inability to secure terms of shipping, insurance and other services needed to facilitate medicines trade [4]. As a result, millions of Iranians that suffer from life-threatening diseases have experienced “exorbitant prices”, stock outs of medicines, and are often forced to purchase drugs from the black market [8].

In Iran, the right to health care and public health services is a constitutional right and is implemented through a network of public providers, the private sector, and NGOs active in health [32]. Iran’s healthcare system is primarily driven by an insurance-based system and has undergone a number of reforms over the last 30 years focusing on expanding primary care coverage (including for rural populations,) the integration of health services and medical education, and improving services in hospital settings [33, 34]. With the second largest population in the Middle East and North Africa, Iran’s growing dual burden of communicable and non-communicable diseases in Iran creates urgency of ensuring access to medicines and illustrates how acute drug shortages disproportionately affect different patient groups in various ways [35].

As an example, HIV/AIDS cases in Iran are rapidly increasing - UNICEF’s 2013 statistics indicate there are a total of 71,000 Iranian HIV/AIDS patients - representing a critical need to ensure adequate access to antiretroviral drugs [36]. Diseases that require expensive and advanced therapeutics to manage chronic or degenerative conditions are also increasingly being diagnosed (e.g., 37,000 Iranian patients suffer from multiple sclerosis.) [37] NCDs are also on the rise with 85,000 patients diagnosed with cancer every year, a phenomenon characterized as a “cancer tsunami” [38, 39]. All of these patients require uninterrupted, sustainable, and safe access to essential drugs.

Consequentially, patients struggling with cancer, multiple sclerosis, blood disorders, and other serious conditions are some of the most negatively impacted by drug scarcities [40, 41]. Medications needed to treat these complicated diseases manufactured by multinational pharmaceutical companies such as Pfizer Inc. (U.S,), GlaxoSmithKline plc (U.K,), and Bayer AG (Germany), are especially hard to find in Iranian pharmacies [42]. Patented medicines originating from these largely U.S. and European producers are hard to substitute, resulting in a lack of availability of drugs that treat specific diseases for which an alternative form of treatment or generic equivalent is not available from foreign suppliers [43]. Most crucially, the complicated and lengthy export approval process and difficulties in trading in Iranian currency have introduced significant disincentives for pharmaceutical companies seeking to supply drugs to Iran. As an example, a $60 million order from an American pharmaceutical company for an anti-rejection transplant drug failed to reach Iran because no bank would facilitate the transaction [44].

Severe medication shortages in Iran are diverse and span several therapeutic classes and disease states. This includes drug shortages for other critical areas of healthcare delivery, including organ transplant drugs, and even vaccine shortages [4, 41]. Other examples include patients suffering from epilepsy who have developed poor drug adherence due to high drug prices and lack of availability [45]. Patients with blood disorders, including over 7000 hemophilic patients and 8000 thalassemia patients in Iran, are also adversely impacted and have developed certain disabilities because antihemophilia drugs and antibleeding agents are in such short supply [46, 47].

Individual patient cases evidencing the human toll of sanctions have also emerged in media reports [48]. One such case involved a 15-year-old boy, Manouchehr Esmaili, who suffered from hemophilia and died of excessive bleeding because his family failed to find the necessary drugs to save his life [49]. Another example includes the routine child vaccine that protects against the bacterium *haemophilus influenzae*, which causes severe pneumonia and meningitis in infants, in critically short supply in Iran [5]. Additionally, patients waiting years to receive a liver transplant had unsuccessful outcomes due to the inaccessibility of anti-rejection medications—yet another of many illustrations of Iran’s inability to import critical medical supplies.

### Medicines manufacturing and supply challenges

The multilateral sanction regime in place prior to the JCOPA effectively cut off Iran’s financial institutions from the rest of the global banking system, and thus negatively affected almost every segment of Iran’s economy, including the health sector [4]. As a result, medication prices increased 30 to 40 % as a result of dollar–currency fluctuations, making vital medications unaffordable even if they are available to purchase on the local market [3]. Individuals who could afford to pay for expensive drugs often stockpiled medications in fear of future drug scarcities, with such practices often resulting in expiry and waste of medications despite nationwide shortages [41]. Hence, the dual specter of drug shortages and price disruptions due to sanctions has fomented a complex medicines-access dilemma that directly impacts patients’ ability to treat and maintain therapy for their health conditions [50].

The negative impact of sanctions blocking financial transactions in the Iranian public sector has also crippled the struggling domestic pharmaceutical industry. A study by the Woodrow Wilson Center estimated that pharmaceutical products importation has decreased 30 % under the round of economic sanctions prior to JCOPA and the export of pharmaceuticals to Iran from the United States was reduced by half from $31.1 million in 2011 to $14.5 million in 2013 [3–5]. Media outlets have reported that Iranian drug companies and importers encounter numerous challenges while trying to stay profitable and keep their production and distribution channels open [44, 48]. Due to financial-sector and banking sanctions that were in place, many Iranian pharmaceutical companies managed their purchases through foreign banks; reportedly only one bank in Turkey was conducting pharmaceutical transactions with Iran because most financial institutions are concerned about potential sanction violations [44].

More than 85 pharmaceutical manufacturers and 20 API manufacturers exist in Iran, although compounding pharmacies started closing in 2014 as a result of shortages [38]. Iran’s national pharmaceutical industry has always played a major role in producing local generic drugs [51]. Domestic manufacturers produced 96 % of generic medicines and relied on imported raw materials and APIs to compound more complex drugs available in Western countries [52]. Despite domestic production, imported medicines nevertheless accounted for approximately 40 % of the total domestic market value in 2012 [38]. Due to increased sanctions, Iranian producers experienced significant disruptions in imported APIs and finished products that forced them to import these products from Indian and Chinese firms [4]. As a result, high-quality medicines reportedly have been replaced with inferior-quality substitution drugs, which presents unknown quality or patient safety issues [4, 53].

Inaccessibility of vital medications and their raw ingredients combined with Iran’s weakening domestic pharmaceutical industry has also resulted in an influx of counterfeit, fraudulent, and substandard medicines into Iran’s health care system. An unregulated black market has developed as a byproduct of drug shortages, introducing medications whose origins and authenticity are often unknown, and has led to expired medications’ distribution and sale, even at potentially very high prices [8]. Hence, the global counterfeit medicines trade, recognized as a serious public health concern, is one that is currently being enabled as a consequence of drug shortages and ongoing Iranian economic sanctions [54, 55].

### Key characteristics of Iran’s drug shortage

**Fig. 2:**
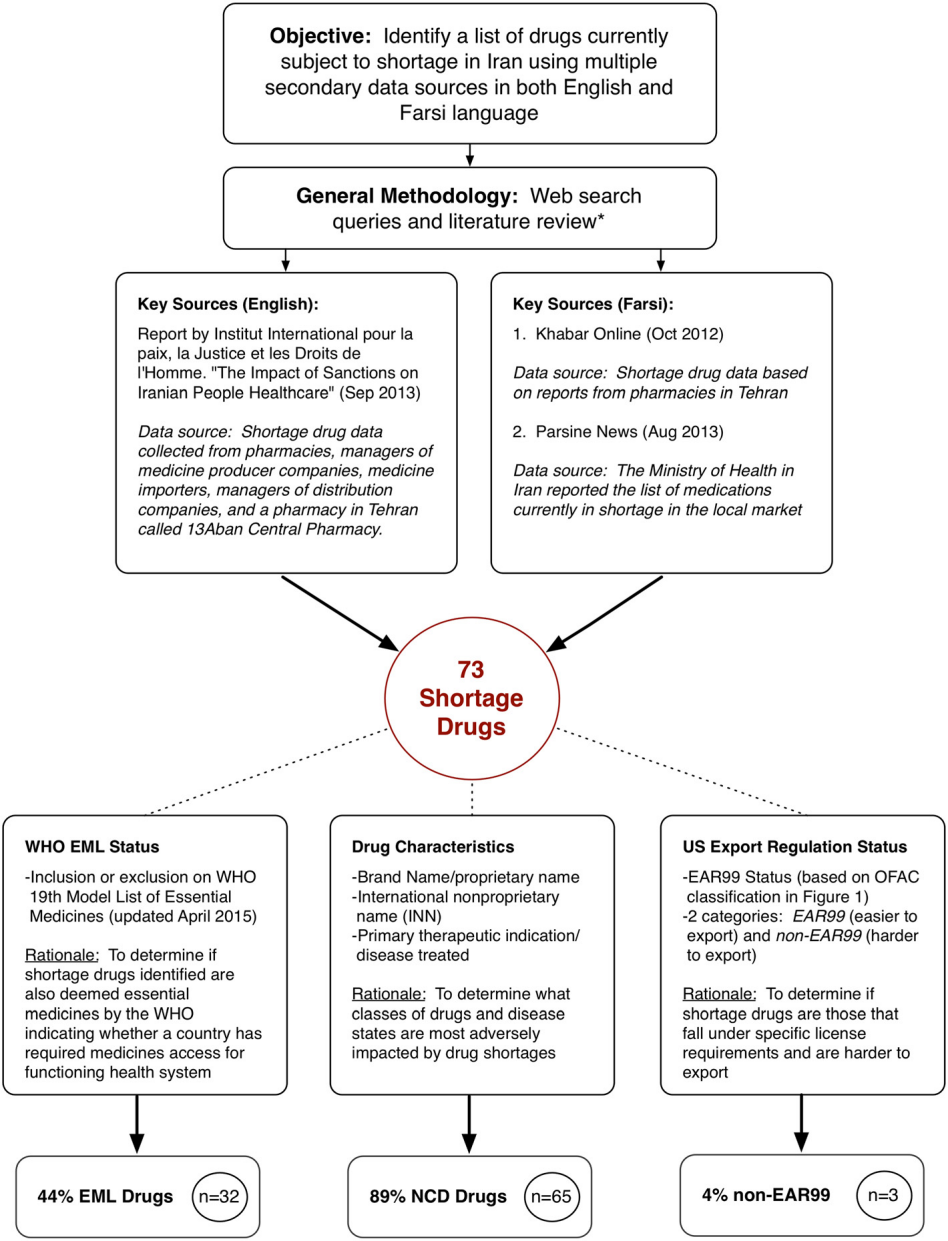
Methodology for drug shortage review

To better understand the scope and magnitude of economic sanctions on the ongoing medicine shortage crisis in Iran, we also examined multiple data sources to identify key characteristics of medicines currently being reported in short supply or unavailable. This was completed by reviewing secondary data sources in both English and Farsi including information in the peer-reviewed literature, technical reports by foundations and non-governmental organizations (NGOs), and information from official Iranian health-related government websites (see more detail regarding the methodology and sources in Fig. 2). Based on these data sources, we compiled a list of drugs identified as in shortage and also characterized: (1) the primary therapeutic indication of the drug; (2) whether the drug was an essential medicine (as determined by the World Health Organizations’ most recent 19th Essential Medicines List, updated April 2015); and (3) whether they would be classified as EAR99 or Non-EAR99. A final list of Iranian shortage drugs and their characteristics is provided in Table 2.

**Table 2:** List of current medications in shortage in Iran

Disease treated	Drug name (INN/Brand name)		EAR status		WHO EML status
			Non-EAR 99	EAR 99	
Diabetes	Glucagon	Glucagon/GlucaGen	Y	N	Y
	Insulin injection	Insulin/ Novofine	Y	N	Y
	Glocophage	Metformin/Fortamet	Y	N	Y
Anticancer chemotherapy	Cytarabine	Cytarabine/Cytosat	Y	N	Y
	Lomustine	Lomustine/Gleostine	Y	N	N
	Doxorubicin	Doxorubicin/Adriamycin	Y	N	Y
	Flurouracil	Flurouracil/Adrucil	Y	N	Y
	MabThera	Rituximab	Y	N	Y
	Chlorambucil	Chlorambucil/Leukeran	Y	N	N
	Xeloda	Capecitabine/Xeloda	Y	N	Y
	Flutamide	Flutamide/Eulexin no equivalency in U.S.	Y	N	N
	Diphereline	Active ingrediate: Triptorelin embonate	Y	N	N
	Tykerb	Lapatinib/Tykerb	Y	N	N
	Leukeran	Chlorambucil/Leukeran	Y	N	Y
	Erbitux	Cetuximab/Erbitux	Y	N	N
	Nexavar	Sorafenib/NexAVAR	Y	N	N
	Thalidomide	Thalidomide/Thalomid	Y	N	N
	Zometa	Zoledronic acid/Reclast	Y	N	N
	Microrelin decapeptyl	Triptoreln/Microrelin Decapeptyl	Y	N	Y
	Infliximab	Infliximab/Remicade	Y	N	N
Anti-asthmatic and chronic obstructive pulmonary disease	Symbicort	Budesonide and Formoterol/Symbicort	Y	N	Y
	Salmeterol	Salmeterol/Serevent diskus	Y	N	N
	Aminophyline	Aminophylline/Phyllocontin	Y	N	N
	Seroflo	Ingredients are Fluticasone and Salmeterol in Peru and Hong Kong	Y	N	N
	Atrovent	Ipratropium inhalation/ Atrovent HFA	Y	N	Y
	Zaditen	ZyrTEC itchy eye	Y	N	N
	Seretide	Advair in U.S. Fluticasone and salmeterol/Advair Diskus	Y	N	N
Cardiovascular medicine	Furosemide	Furosemide/Lasix	Y	N	Y
	Amiodarone	Amiodarone/Cordarone	Y	N	Y
	Flecainide	Flecaidide/Tambocor	Y	N	N
	Lisinopril	Lisinopril/prinivil	Y	N	N
	Sotahexal (available in Poland)	Sotalol/Betapace	Y	N	N
Multiple sclerosis (M.S.)	Ziferon Manufactured in Iran	Interferon beta-1b/ziferon	Y	N	N
	Extavia	Interferon beta-1b/ Betaseron	Y	N	N
	Betaferon	Active substance interferon beta-1b	Y	N	N
	CinnoVex	Active substance interferon beta-1b (manufactured in Iran)	Y	N	N
	Avonex	Interferon beta-1b/Avonex	Y	N	N
Radiocontrast media	Iopromide	Iopromide/Ultravist	Y	N	N
	Iodixanol	Iodixanol/Vasipaque	Y	N	N
	Scanlux	Active ingredient in iopamidol, available in Greece, Spain, Switzerland, Bulgaria, Italy, Hungary and Tunisia	Y	N	N
	Omnipaque	Omnipaque 180, 240, 300 /iohexol	Y	N	Y
Antidote/haemoglobinopathies	Deferoxamine	Deferoxamine/Desferal	Y	N	Y
	Tegretol CR	Carbamazepine/Carbatrol	Y	N	Y
	Sodium valproate	Sodium valproate	Y	N	Y
	Depakin	Active substance: valproic acid sodium available in Italy and Turkey	Y	N	Y
	Orlept	Active substance: Valporic Sodium Available in U.K.	Y	N	Y
	Exjade	Deferasirox/Exjade	Y	N	Y
Antiviral	Ganciclovir	Ganciclovir/Cytovene	Y	N	N
	Tenofovir	Tenofovir/viread	Y	N	Y
Antidepressant	Asentra Available in listed countries: Latvia, Poland, and Serbia	Active ingredient sertraline	Y	N	N
	Doneurin available in Germany	Doxepin	Y	N	N
	Doxepin	Doxepin/SINEquan	Y	N	N
	Sertraline	Sertraline/Zoloft	Y	N	N
Infertility treatment	Human Chorionic Gonadotropin (HCG)	HCG/Novarel	Y	N	N
	Cetrorelix	Cetrorelix/Cetrotide	Y	N	N
Pregnancy termination	Misoprostol	Misoprostol/Cytotec	Y	N	Y
Antiparkinsonism medication	Madopar available in U.K.	Levodopa and Benserazide	Y	N	N
	Levodopa	Levodopa/Larodopa	Y	N	Y
Antibacterial Medication/Antibiotics	Azithromycin	Azithromycin/ Z-pack, Zmax	Y	N	Y
	Klacid available in Australia	Klacid/Clarithromycin	Y	N	Y
Antimalarial	Pyrimethamine	Pyrimethamine/Daraprim	Y	N	N
Transplant	CellCept	Mycophenolate mofetil/ CellCept	Y	N	N
Anticoagulant	Warfarin	Warfarin/Coumadin	Y	N	Y
Vaccines	Influenza vaccine	Influenza virus vaccine/ Afluria	N	Y	Y
	BCG (Bacillus Calmette-Guerin)	BCG/TheraCys	N	Y	Y
	Gardasil	Human Papillomavirus Vaccine (HPV)/Gardasil	N	Y	Y
ADHD treatment	Ritalin	Methylphenidate/Concerta	Y	N	N
Alzheimer’s disease	Galantamine	Galantamine/Razadyne	Y	N	N
	Reminyl	Reminyl/galantamine hydrobromide	Y	N	N
Prevention of endometrial hyperplasia	Progesterone	Progesterone/Prometrium	Y	N	N
	Cyctogest	Cyclogest 200/progestron	Y	N	N
Antiepileptic/Anticonvulsant	Tegretol CR	Carbamazepine/Carbatrol	Y	N	Y
	Sodium valproate	Sodium valproate	Y	N	Y

Based on this review, we identified 73 drugs reported as subject to shortage in Iran (referred to further as “shortage drugs”). After examining the therapeutic classifications of shortage drugs, it was determined that 89 % (*n* = 65) were for medications used to treat NCDs, and the remaining 11 % (*n* = 8) addressed infectious diseases. Of the 65 shortage drugs treating NCDs, 44 % (*n* = 32) were used to treat the four most prevalent NCDs in the Iranian population (based on WHO data,) including cancer (23 %, *n* = 17), respiratory disease (10 %, *n* = 7), cardiovascular disease (7 %, *n* = 5), and diabetes (4 %, *n* = 3). The remaining 45 % (*n* = 33) of shortage medications were used to treat a variety of other NCDs including hemophilia, Parkinson’s disease, Alzheimer’s disease, multiple sclerosis, fertility issues, various mental health issues (antidepressants), Attention Deficit Hyperactivity Disorder, and transplant drugs. The eight identified shortage drugs that were used to treat infectious diseases included treatments for malaria, tuberculosis, and HPV.

To assess whether shortage drugs represented therapies needed to treat conditions and diseases with a high disease burden in Iran, we compared the therapeutic classification of identified shortage drugs to data contained in WHO’s non-communicable disease country profiles [56]. According to data available from 2014, the leading cause of death for Iran’s population of 76 million people (395,000 reported deaths) was NCDs, which accounted for 76 % of all patient mortality. The mortality breakdown for NCDs in Iran comprised of: cardiovascular disease (46 %), cancers (13 %), other NCDs (11 %), and chronic respiratory disease and diabetes (both 2 % respectively). In comparison, communicable, maternal, perinatal and nutritional conditions comprised a total of 10 % of mortality [56].

**Fig. 3:**
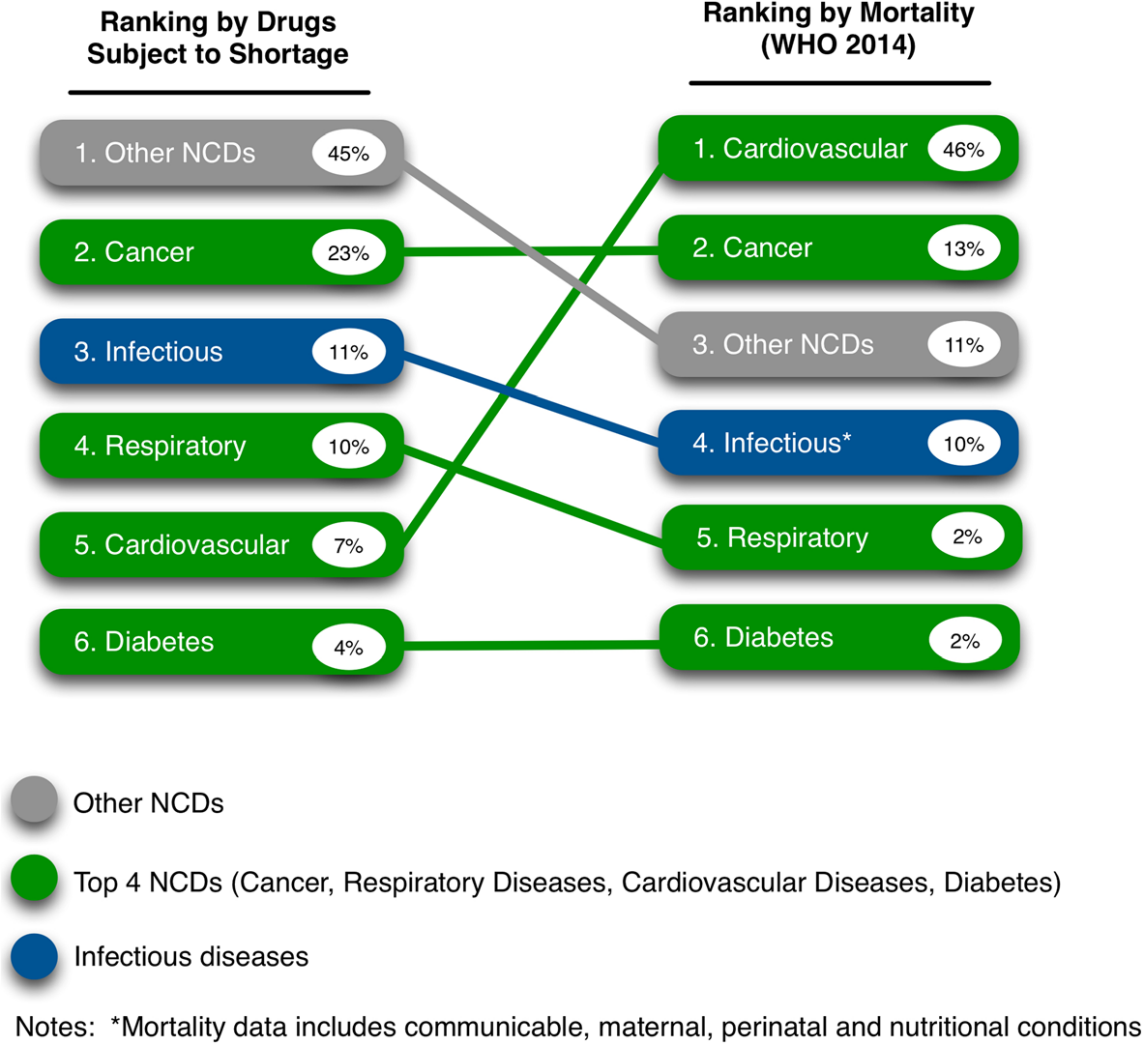
Shortage drug ranking and mortality ranking* (*excluding road accidents and injury)

When comparing the aforementioned shortage drug rankings to WHO mortality data based on therapeutic class, we noticed similar distributions. Among therapeutic drugs, other NCDs ranked first among the shortages, specifically medications used to treat multiple sclerosis, Alzheimer’s, Parkinson’s disease, hemophilia, thalassemia, and depression. Cancer medications ranked second, infectious-disease medications ranked third, respiratory disease medications ranked fourth, cardiovascular medications ranked fifth, and diabetes medications ranked sixth. A similar pattern was observed in the WHO mortality rate chart: the major leading causes of death were cardiovascular disease, ranked first and road accidents and injuries ranked second (the only significant deviation.) Cancer was the third leading cause of death in Iran, followed by other NCDs which ranked fourth, infectious diseases ranked fifth, and respiratory disease and diabetes ranked sixth (See Ranking Comparison, Fig. 3). These comparisons indicate that the ranking of shortage drugs in Iran tracks reasonably well with the overall disease burden (specifically disease-related mortality) of the Iranian population.

We then cross-referenced shortage drugs with information from the WHO Essential Medicines List (19th Edition), which yielded identification of a subset of 32 shortage drugs (44 %) that are also categorized by the WHO as essential medicines. The EML is a critical instrument that informs medicines’ selection in national formularies and is also an effective tool in assessing if a given population has access to essential medicines, which is a fundamental right generally recognized in international health and human rights law [57]. This finding indicates that close to half of all drugs subject to shortage in Iran are necessary and crucial to ensure a functioning public health system. Finally, we assessed EAR classifications and found that 70 shortage drugs (96 %) were EAR99–classified, fall under the general OFAC license, and should in theory be easier to export when compared to non-EAR99 drugs. Conversely, we identified only three shortage drugs (4 %) as non-EAR99–classified drugs that are harder to export because they require greater effort to secure export clearance under the current OFAC regulations.

## Discussion

Based on our examination of the history of the multilateral economic sanctions regime against Iran, current regulatory requirements for exporting medicines to Iran, and the characteristics of drugs currently in shortage, it is clear that the public health consequences of economic sanctions have not been appropriately addressed to ensure equitable and safe access to essential medicines. This is despite the fact that Iranian sanctions have historically been characterized as “smart” and “targeted,” and argued as primarily aimed at the Iranian government and its leaders as a diplomatic tactic to curb nuclear proliferation activities, not intended to harm the civilian population. However, the reality of the situation for most ordinary Iranians is that ongoing medicine shortages are denying access to needed treatment for debilitating and often life-threatening diseases. This brings into question whether economic sanctions are violating internationally-agreed upon principles of the human right to health and whether, in the context of their expansive nature and impact far beyond nuclear proliferation activities, they have a sufficient legal basis under international law [26, 58].

Additionally, our review of current acute medicine shortages in Iran also indicates there is a possible “three-pronged effect” that directly negatively impacts public health outcomes in Iran. First, the therapeutic characteristics of shortage drugs generally align with disease-related mortality in the country, inferring that shortages may be exacerbating the country’s overall disease burden. The human toll of these shortages is undeniable, given multiple reports of vulnerable Iranian patients suffering from lack of treatment and poor health outcomes, including children, women, the elderly, and patients with advanced diseases [4, 59]. Second, almost half of the drugs in short supply are deemed “essential” by the WHO, indicating that Iran’s health system lacks sufficient access to drugs that are the minimum of what is required to ensure a functioning health system. Third, 96 % of the drugs in shortfall are EAR99–classified medicines that technically should be easier to export to Iran but nevertheless remain in critical shortage despite regulatory changes to facilitate humanitarian trade in U.S. export regulations. This signals that OFAC’s revisions and recent efforts to ease sanctions for humanitarian reasons may not be effective in addressing the underlying factors that restrict trade in medicines: primarily the complexity of the export licensing regime and financial restrictions on the operational banking system that continue to create significant disincentives for companies seeking to supply life-saving medications to Iran [4].

Given these findings and the limitations of current policy and regulatory approaches, it is clear that additional health advocacy and diplomacy are needed to address this acute and underserved public health crisis. Herein lies the opportunity to leverage the historical opportunity presented by the 2015 international negotiation of the JCPOA, by inserting this critical public health issue into ongoing discussions of implementing the various stages of the agreement. This should include advocating for the tangible application of “global health diplomacy”, generally defined as diplomatic activities that prioritize global health issues in the foreign policy context, in order to ensure that ongoing multilateral negotiations do not neglect the public health and humanitarian needs of Iranians to gain access to life-saving and essential medicines [60]. Specifically, the historic JCPOA agreement can serve as a facilitating mechanism to integrate public health objectives into foreign policy through applied health diplomacy by directly incorporating health and humanitarian conditions into the construction and implementation of the agreement moving forward. As currently constructed, the JCPOA does not contain any specific mechanisms that ensure greater access to life-saving drugs or measures to ensure that sanctions do not continue to harm the health of the Iranian people, but does contain provisions that can be built upon for this goal.

Specifically, following commencement of “Implementation Day”, OFAC issued an updated guidance document that outlines the U.S. Government’s commitment and steps taken towards lifting nuclear-related sanctions on non-US persons and companies in several sectors including the banking, finance, energy, trade, transport, raw materials, and energy industries consistent with the terms of the JCPOA [61]. This included removing over 400 individuals from the SDN List and other sanctioned lists of persons (which will also allow these entities to reconnect to the global banking system via SWIFT) and gave Iran access to an estimated $60 billion in foreign exchange reserves that were previously frozen [61].

However, though the guidance supersedes several provisions of U.S. sanction legislation and Executive Orders, it leaves in place the broader U.S. domestic trade embargo on Iran and generally continues to prohibit U.S. entities from engaging in transactions or dealings with Iran unless these activities are specifically exempt or authorized by OFAC regulations [61]. The EU has also followed suit, although has more broadly lifted economic and financial sanctions in connection with the Iranian nuclear program, including allowing economic and trade activity directly for EU persons and entities for the finance, banking, insurance, energy, transport, and other sectors [62].

Though the JCOPA represents a critical pathway forward paving the way for Iran’s reintegration in to the global economy, international businesses may continue to take a cautious approach. This is largely due to the fact that U.S. banks remain prohibited from engaging in direct transactions with Iran, limiting the country’s access to US dollar-denominated transactions, a condition that will continue to disincentivize medicines procurement from U.S. pharmaceutical companies [63]. There is also confusion regarding how consistently sanction relief is being applied, with 86 % of the Iranian entities on the UK HM Treasury sanction list being removed compared to only 68 % on the US OFAC sanctions list [64].

In addition, the terms of the JCOPA also allow nuclear-related sanctions pre-JCOPA to “snap back” or be reinstated in the event of Iran’s failure to comply its international commitments. These provisions may create an undesirable business environment for potential trading partners and foreign banks, as a “snap back” of sanctions would effectively negate any investments made in financial operations made in Iran [63]. Further, the Financial Action Task Force, an international body for anti-money laundering and terrorist financing rules and regulations, recently issued a statement that it remained “exceptionally concerned” about the Iranian banking system, reflecting ongoing concerns about the current and future role of Iran in the international financial system [65]. This continued scrutiny of Iran’s finance system, combined with the fact that the country’s banking infrastructure is seriously outdated, may make doing business with Iran unattractive despite recent sanctions relief under the JCOPA [63].

Hence, to address these policy gaps that continue to persist in the Iranian sanctions regime and the construction of the JCOPA, we recommend a set of additional policy measures that can be built into the JCPOA’s non-nuclear sanctions relief and phasing plan with the definitive aim of alleviating the current Iranian medicines shortage. Our recommendations focus on addressing underlining challenges as already identified including: establishing regulatory export harmonization; amending the OFAC EAR99 classification system to make it easier for U.S. companies to export medicines; exempting vaccine products from stringent export controls; allocating a protected SWIFT line specifically for humanitarian medicines trade; providing additional clarification that Iranian oil revenues can be freely used for medicines procurement without reservations; and exempting medicine and medical commodities from “snap back” provisions (see Table 3). These policy measures need to be acted upon before the next JCPOA milestone, “Transition Day,” which is still some 8 years away.

**Table 3:** Policy proposals for improving access to medicines in JCPOA

Topic	Description
Regulatory sanction harmonization	Existing contradictions in various U.S. and European sanctions must be resolved to harmonize the process of permitting humanitarian transactions to take place. Most importantly, the United States must make it unambiguously clear that both U.S. and financial institutions from other countries are fully authorized to transfer funds in support of procuring and supplying humanitarian medical goods to Iran that are not classified as “dual-use” commodities.
OFAC classification	The U.S. government should revisit its OFAC classification process for non-EAR99 medications and exempt specific products that meet critical Iranian population health needs, including those drugs, medical devices, and diagnostic products which address diseases with high mortality rates or disease burden. Additionally, OFAC should specify medical products that are non-EAR99–classified in lieu of listing broad categories of products.
Vaccines	OFAC should specifically remove vaccine products from the Non-EAR99–classified drugs list and add them instead to the EAR99–classified drugs list because they are crucial tools in disease prevention and public health outcomes.
SWIFT line	All international partners should allocate a dedicated SWIFT line to transfer funds for medical purposes and designate certain Iranian and foreign banks as specifically authorized to transfer funds for these medicines and medical devices. This would be similar to proposed OFAC SWIFT line that will be dedicated for medicine and medical devices purchases following the JCOPA.
Oil revenues	Provide definitive clarification of the terms for waivers/exemptions for purchasing Iranian crude oil in a way ensures trading partners that Iran can access to its oil revenues deposited in foreign banks and allow the currency to be used for life-saving medicines to be purchased from U.S. and EU companies.
Exemption from “Snap Back” provisions	Policy proposals above should be exempt from “snap back” provisions in the event of non-compliance to the terms of the JCPOA. This will ensure reliable and ongoing access to life-saving treatments and provide trading partners and banking institutions with confidence to invest in medicines procurement.

Equally critical is ensuring that the deleterious impacts of economic sanctions on human health as have occurred due to Iran’s drug shortages are not repeated in the future. This should include advocating for the integration of Health Impact Assessments (HIAs) that identify the health consequences of sanctions while also ensuring proper planning, monitoring, and implementation to prevent or mitigate potential negative effects on population health [66, 67]. This could be accomplished by establishing procedures that require an HIA to be carried out by the UN Security Council for any economic sanctions supported by a UN resolution or enacted by its permanent and non-permanent Security Council members. These HIAs could be carried out independently by the WHO or other public health organizations, and would allow for scientific evaluation to determine if sanctions violate the health and human rights of communities, while also focusing on improving health outcomes through policy change that could translate into action during diplomatic negotiations.

## Conclusions

Clearly, the decades-long economic sanctions regime has had a severe, detrimental public health impact and led to poor health outcomes among Iranians. Although the economic sanctions may have had their intended effect of bringing Iran to the negotiating table and ending decades of diplomatic impasse between Iran and the West regarding nuclear proliferation, the unintended and serious public health consequences of sanctions have yet to be addressed sufficiently. The expansion of economic sanctions against Iran have played a key role in creating a medication shortage that directly impacts ordinary Iranian citizens and arguably denies their human right to health. Further, revisions to regulatory processes and actions taken under the JCOPA do not appear to be sufficient, as ongoing concerns about the ability to facilitate financial transactions continue to make it difficult for Iranian health care providers and their patients to access essential medicines. As in all other countries, an undisrupted supply of safe and affordable medicines originating from domestic manufacturing and imports is vital to a functioning health system. Acknowledging this fundamental need, we argue that the time is now for a practical application of health diplomacy that takes advantage of the historical JCPOA agreement with the aim of finally addressing the immediate humanitarian and public health needs of the people of Iran and what should be their fundamental right to access safe medicines.
